# Validity and Reliability of the Portuguese Version of the Connor–Davidson Resilience Scale of 10 Elements for Young University Students

**DOI:** 10.3390/healthcare12030400

**Published:** 2024-02-04

**Authors:** Lorena Tarriño-Concejero, Dalila Cerejo, María Dolores Guerra-Martín, Juan Manuel Praena-Fernández

**Affiliations:** 1 Faculty of Nursing, Physiotherapy and Podiatry, University of Seville, 41009 Seville, Spain; ltarrino@us.es; 2 Institute of Biomedicine of Seville (IBiS), 41013 Seville, Spain; 3 Department of Sociology, Faculty of Social Sciences and Humanities (NOVA FCSH), Interdisciplinary Centre of Social Sciences (CICS.NOVA), Nova University Lisbon (NOVA), 1069-061 Lisbon, Portugal; 4 Faculty of Medicine-Biostatistics Unit, University of Granada, 18016 Granada, Spain; jpraena_2@ugr.es

**Keywords:** COVID-19, mental health, resilience, psychological, students, validation study

## Abstract

Background: Resilience is an important aspect of mental health in young people, which has become more relevant after the COVID-19 pandemic. It is therefore of paramount importance to have valid and reliable instruments that measure the globality of this aspect. One of the instruments that has been shown to have good psychometric properties and which has been widely adapted in several languages is the Connor–Davidson resilience scale, composed of 10 elements (10-item CD-RISC). Aim: The aim of this study was to evaluate the validity and reliability of the Portuguese version of the 10-item CD-RISC among young university students. Methods: a cross-sectional observational study of psychometric validation was conducted with a sample of 206 university students. Results: Good and adequate fit indices were obtained for the confirmatory factor analysis (CFA): Standardized Root-Mean-Square Residual [SRMR] = 0. 056; comparative fit index [CFI] = 0.958; and the Tucker–Lewis index [TLI] = 0.946. It also showed an average degree of convergent validity with the Depression, Anxiety and Stress Scale (DASS-21) and the General Health Scale (SF-36), and its internal consistency was good (Cronbach’s alpha = 0.842) with a range of factor loadings between 0.42 and 0.77. Conclusions: the results show that the 10-item CD-RISC is a valid, reliable scale to measure resilience among young Portuguese university students.

## 1. Introduction

Resilience is of considerable importance in the study of a number of health problems, and in recent years, due to the COVID-19 pandemic, it has been used in research in numerous countries as a means of helping detect and identify factors that contribute to improving the health of a community in general [1,2].

Resilience can be defined as a protective factor against mental problems and as a dynamic process of adaptation to changes in life circumstances [3]. From a positive psychological viewpoint, it is positive growth or adaptation after periods of biopsychospiritual homeostatic interruption, focusing on strengths that allow individuals to survive and grow even in the face of adversity, and not just a simple mechanism or process of recovery from a stressful situation, as proposed from a psychopathological perspective [4,5]. It is a multidimensional characteristic that varies depending on the cultural origin, context, personal circumstances, time, age, and gender of the individual [5]. 

Resilience research emerged in 1982 in a multiracial population of children to observe the risk factors that led to psychosocial problems and identify the individuals’ strengths [6,7]. Since then, the field of research into resilience has evolved mainly through three stages [8]. The first phenomenological stage is based on understanding what situational traits or premises make people possess the strengths to survive adversity. The second stage is focused on the process of discovering how a person acquires the characteristics that make them resilient. Finally, the third stage is based on designing models to develop a theory and an exact definition of resilience [8].

In this last stage, there have been a considerable number of publications of studies focused on identifying and measuring resilience by designing valid and reliable measurement instruments for the target population. Thus, in recent years, different systematic reviews have been published which aim to identify, compare, and critically evaluate the validity and psychometric properties of conceptually similar scales and make recommendations on the most appropriate use for a specific population, intervention, and outcome. Among these, a review focusing on people over 60 years of age is of particular interest [9], together with others dealing with family resilience [10], indigenous adolescents [11], and the general population [12]. These reviews have included and analyzed a large number of studies using different instruments such as the Connor–Davidson Resilience Scale, Wagnild and Young’s Resilience Scale, the Brief Resilient Coping Scale, the Dispositional Resilience Scale, the Psychological Resilience Scale, the Adolescent Resilience Scale, and the Resilience Scale, among others. Some of these reviews propose that one of the most appropriate instruments is the Connor–Davidson Resilience Scale (CD-RISC) due to the good psychometric properties shown in relation to its structural validity, internal consistency, reliability, and cross-cultural validity [5]. 

The CD-RISC is a 25-item self-administered scale that assesses various aspects of resilience, including self-efficacy, tolerance to negative effects, positive acceptance of change, and perceived social support. It has demonstrated excellent structural validity based on five factors: ‘personal competence, high standards, and tenacity’; ‘trust in one’s instincts, tolerance of negative affect, and the strengthening effects of stress’; ‘positive acceptance of change and secure relationships’; ‘control’; and ‘spiritual influences’ [5]. A shorter, 10-item version with a single dimension, which is easier to use in both clinical and community contexts, has also been validated [4]. Both versions have been validated in various countries, such as the United States, China, Japan, Korea, Portugal, Brazil, Australia, the United Kingdom, Iran, and Spain, among others, showing good psychometric properties [13]. 

In Portugal, the 10-item CD-RISC was validated in a sample of adults in employment centers, showing good psychometric properties [14], although it has not yet been validated in the young Portuguese student population.

### The Present Study

This study aimed to validate the 10-item CD-RISC in the young Portuguese student population. Previous research on scales that measure resilience has shown that the CD-RISC is one of the most reliable instruments measuring resilience. Although this scale has already been validated within the same culture, in Portugal, among adults, this population differs greatly from the patterns of behavior and economic and social circumstances of young people. Therefore, it is necessary to test whether the scale obtains good psychometric properties in the sample that is the object of our research.

Additionally, instruments are needed to measure resilience in a university context. This is a space where young people socialize, interact, and spend a large part of their time. Moreover, the university years take place in a crucial phase of development known as emerging adulthood [15], which coincides with the peak of the onset of many mental and behavioral disorders [16,17]. Universities must also play an active role in promoting the health and well-being of both their students and their staff and society in general, leading and supporting processes of social change [18,19]. In fact, Portugal is affiliated with the Ibero-American Network of Health Promoting Universities (RIUPS), whose commitment is to give greater priority to health promotion, integrate health in a transversal way in its policies, and create healthy university plans [20]. It is also clear that the COVID-19 pandemic has marked a turning point in university community strategies and has highlighted the need for research studies to be carried out in this field.

Thus, firstly, we predicted that the factorial structure of the 10-item CD-RISC in young Portuguese university students would confirm the one-dimensionality proposed by the original scale. Second, we predicted that resilience would have a strong, positive relationship with better states of overall health. Finally, we predicted that resilience would correlate strongly and negatively with depression, stress, and anxiety.

## 2. Materials and Methods

### 2.1. Research Design

**Figure 1:**
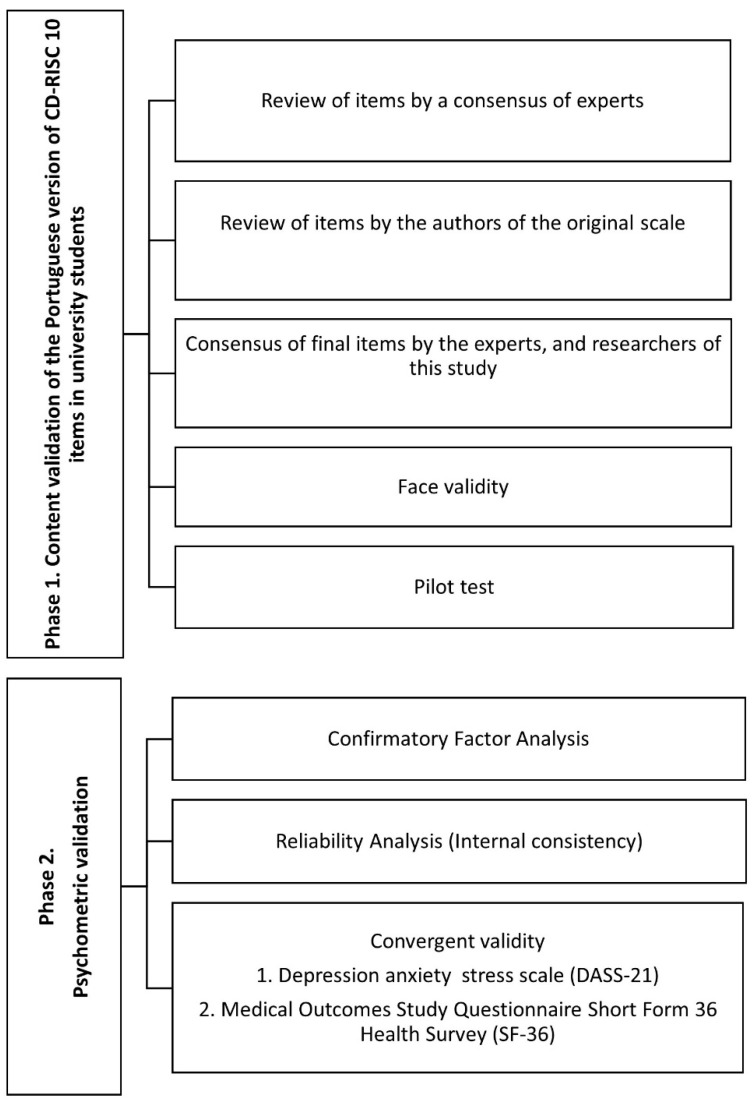
Phases of the research.

The 10-item CD-RISC was validated in the Portuguese university population using a quantitative cross-sectional psychometric validation design. In the first phase, the items were subjected to a consensus of experts, facial validity, and piloting. In the second, the psychometric properties of the 10-item CD-RISC were analyzed (Figure 1). The Consensus-Based Standards for the Selection of Health Measurement Instruments (COSMIN) Study Design checklist was followed for this study [21].

### 2.2. Procedure and Participants

The data used are part of a larger dataset obtained through research on dating violence and its relationship to mental health and resilience. 

The sample was obtained using criteria for instrument validation proposed by Mokkink et al., 2019 [21], where the sample size must be at least 7–10 subjects per item. The sample size was calculated based on the total number of students enrolled in degrees at the Faculty of Social and Human Sciences of the Nova University of Lisbon (n = 2680), academic year 2021–2022, with a confidence level of 95% and a precision (margin of error) of 7%, obtaining an estimated sample size of 184 students. The sample was selected using a non-probabilistic convenience sampling of students from all courses, whose inclusion criteria were to be enrolled in a degree at the faculty, to be between 18 and 24 years old, and to have linguistic competence in Portuguese to sufficiently understand the instrument. 

A self-administered online survey using the Google Forms platform was used, which was provided through “Inforestudante”, the university’s digital platform. The study was conducted between July 2021 and February 2022. 

### 2.3. Measures

#### 2.3.1. Demographic Variables

The sociodemographic data collected were related to age; sex; area of origin (rural or urban); whether they were in active employment and, if so, the number of hours per week they worked; and whether they received social support (financial aid for studies) from their university.

#### 2.3.2. Resilience

Resilience was evaluated using the 10-item CD-RISC validated for Portuguese adults in employment centers [14]. It is a self-administered, 10-item, single-disciplinary questionnaire using a Likert-type scale with five answer options (0 = never; 4 = almost always). Higher scores indicated a higher level of resilience. Acceptable indices of adjustments were obtained in the confirmatory factor analysis (CFA), as well as adequate internal consistency (0.91–0.89) for the different groups analyzed [14]. To adapt the items to the young Portuguese university population, consent was first requested from the author of the original English version, and an agreement was signed authorizing us to use and validate the new version of the scale. Second, the items were submitted to a panel of two experts in mental health, resilience, and youth. In the first round, two of its items were proposed for minor modifications. The author of the original version was then consulted, and the new version was compared with other versions validated in the same population in different locations: the USA, Canada, Korea, Singapore, Spain, the Netherlands, India, Hungary, Italy, Nigeria, and Australia [13]. The aim was to maintain the same homogeneity in the items across all countries, and the experts and researchers of this study reached a second consensus with the author of the original version not to modify the items since the suggested changes were minimal. 

Later, these items were subjected to face validity tests with 25 young university students to examine their clarity, accuracy, and comprehension. All the items were found to be clear, accurate, and well understood, with mean scores between 3.57 and 3.76 on a scale of 1 to 4. According to the criteria of Abad et al., 2011 [22], 3 items must be accepted without modification. Subsequently, all the items were subjected to a pilot study and an analysis of their psychometric properties.

#### 2.3.3. Health-Related Quality of Life 

The Short Form 36 Health Survey of Medical Outcomes Study Questionnaire (SF-36) was used to examine health-related quality of life. This scale evaluates 8 dimensions of health, physical function, physical role, body pain, general health, vitality, social function, emotional role, and mental health, which are measured through 36 items. Each dimension obtains scores between 0 and 100, where 0 is the worst state of health and 100 is the best state of health related to each of the dimensions. This scale has been validated in Portugal, obtaining good psychometric Cronbach’s alpha properties for the different dimensions between 0.645 and 0.875 [23].

#### 2.3.4. Depression, Anxiety, and Stress

This variable was assessed using the 21-item depression, anxiety, and stress scale (DASS-21), validated in Portugal [24], which shows the same factorial structure as the original version: 21 elements making up 3 scales (depression, anxiety, and stress) with 7 elements each. Each element evaluates the degree to which the subjects experienced each symptom during the past week on a 4-point severity or frequency scale: 0 = did not apply to me at all, 1 = applied to me to some degree, or some of the time, 2 = applied to me to a considerable degree or a good part of time, and 3 = applied to me very much or most of the time. The highest scores on each scale correspond to more negative affective states. Good psychometric properties and a Cronbach’s alpha of 0.85 were obtained for the depression scale, 0.74 for anxiety, and 0.81 for stress [24]. 

All these measures were included in the final questionnaire, and a pilot test was carried out before moving on to the phases of analyzing the psychometric properties with the aim of reducing possible biases and errors in obtaining subsequent data [25]. A sample of 32 university students participated in this pilot survey, and the overall questionnaire was understood and seen to be adequate, and no errors were detected.

### 2.4. Data Analysis

For the univariate data analysis, we calculated means and standard deviations (SDs) for the quantitative variables and absolute and relative frequencies for the qualitative variables. In the bivariate analysis, normality was previously calculated using Kolmogorov–Smirnov tests as the data turned out to follow normality, and the Student’s *t*-test was used for the difference in sex between the mean scores of the 10-item CD-RISC. Finally, the U-Mann–Whitney test was used for the items. 

The psychometric analysis included validity and reliability tests. First, to verify the validity of the construct, an exploratory factor analysis (EFA) was performed using the principal component Varimax and the rotation analysis method. The Kaiser–Meyer–Olkin (KMO) test and Bartlett’s sphericity test had already been performed to determine whether the data were suitable for a factor analysis. A sedimentation graph was used to analyze the suitability of the number of factors extracted. Subsequently, the one-dimensional structure was confirmed by a CFA using the robust maximum likelihood estimation (MLM) method, with the following fit indices (FI): the Standardized Root-Mean-Square (SRMR), the comparative fit index (CFI), and the Tucker–Lewis index (TLI). A good model fit was indicated by SRMR values < 0.05 and CFI and TLI values ≥ 0.95 [26], and the ratio of the chi-square to the number of degrees of freedom (χ2/DF) was calculated, which was considered acceptable if the resulting value was higher than 3 [27].

To assess convergent validity, we compared the 10-item CD-RISC scores with the DASS-21 and the SF-36 using Pearson’s correlation coefficient, where we anticipated a strong positive correlation with the SF-36 and a strong negative correlation with the DASS-21. For the interpretation of the values, we followed the criterion of Hernández et al., 2010 [28], where correlations within a range between 1.00 and 0.91 are perfect, between 0.90 and 0.76 are very strong, between 0.75 and 0.51 are considerable, between 0.50 and 0.11 are medium, between 0.10 and 0.010 are weak, and 0.00 indicates no correlation.

Reliability was assessed by internal consistency using Cronbach’s alpha test, with an acceptable value of >0.7 [29].

All analyses were performed using SPSS version 26, R version 3.6.3, and AMOS version 23.

### 2.5. Ethical Considerations

This research was approved by the research support division of the Nova University of Lisbon (Portugal), with approval number 1/CE_NOVAFCSH/2021. The entire study complied with the Declaration of Helsinki on the ethical protection and regulation of research among human beings. The participants received an informative letter explaining the nature of the investigation as well as the voluntary nature of their participation and what was expected of them. Subsequently, those who wanted to participate signed an informed consent form acknowledging their intention to participate and stating that they understood the characteristics of the study. The data were anonymized to ensure the anonymity of the participants. For the data analysis, only two researchers accessed the data for added security. The data were kept under lock and key in the Department of Nursing of the University of Seville once downloaded from the Google Forms platform.

## 3. Results

### 3.1. Demographic Characteristics and Scores of the 10-Item CD-RISC Scale in the Sample

The sample consisted of 206 participants, 26.69% men and 73.31% women, with a mean age of 21.10. In addition, 92.2% lived in urban areas and 7.8% in semi-urban areas, and 22.8% worked an average of 6.25 h (SD 12.43). Only 24.8% received social support (financial aid for studies, transport, accommodation, and/or food) from their university.

The mean score for resilience for the total sample was 23.50 (SD 6.92), with men scoring higher (M = 26.22, SD = 6.05) compared to women (M = 22.51, SD = 6.97), with a statistically significant difference (*t* = 3.495, *p* = 0.001) and an average effect size of d = 0.55. The results are shown in Table 1.

### 3.2. Construct Validity

**Figure 2:**
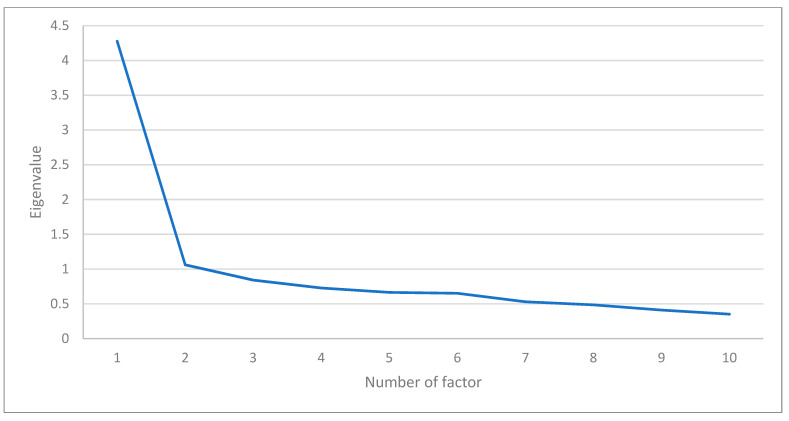
Sedimentation graph of factor components of the 10-item CD-RISC, Portuguese version, among young university students.

The EFA was performed using the principal component Varimax and the rotation analysis method. The Kaiser–Meyer–Olkin measure (KMO = 0.893) and Bartlett sphericity test (X2 = 624.49; df = 45; *p* = 0.001) confirmed that the model was suitable for the data. One component was extracted by the Kaiser criterion and confirmed by the optimal number of factors graph. This one-dimensional solution had an explained variance of 42.78%. The sedimentation graph showed a single suitable factor solution (Figure 2).

**Figure 3:**
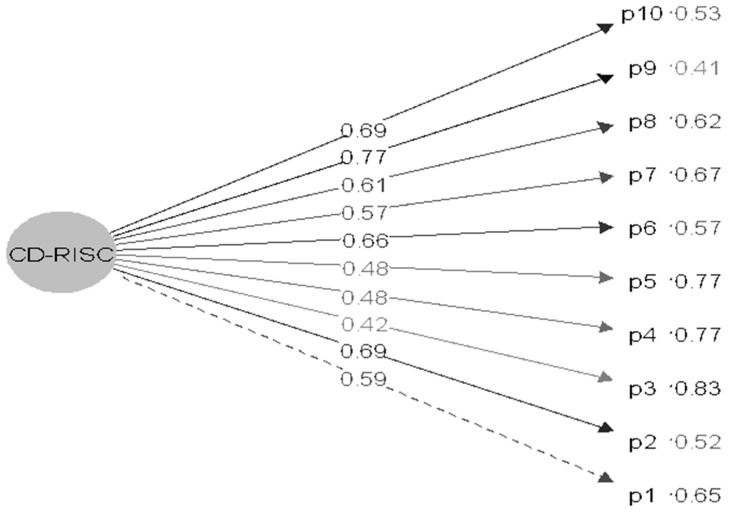
CFA diagram of the 10-item CD-RISC, Portuguese version, among young university students.

Subsequently, the CFA confirmed the one-factor structure, showing good adjustment indices: X2 = 94.642 (*p* = 0.000; df = 35); SRMR = 0.056; TLI = 0.946; and CFI = 0.958. The factorial loads of the items ranged from 0.42 to 0.77 (Figure 3).

### 3.3. Convergent Validity

We compared the mean scores of the 10-item CD-RISC scale with the mean scores of the different dimensions of the DASS-21 and SF-36 scales. All correlations were statistically significant (*p* < 0.01). The CD-RISC showed a negative correlation in the middle range of the DASS-21 scale with depression (r = −0.454, *p* < 0.01), anxiety (r = −0.331, *p* < 0.01), and stress (r = −0.340, *p* < 0.01). These results confirmed the previously stated hypothesis that higher resilience scores would correlate with lower stress, depression, and anxiety scores. On the other hand, the 10-item CD-RISC was positively correlated in the middle range with all dimensions of the SF-36 scale, showing stronger correlations with three of its dimensions, general health (r = 0.447, *p* < 0.01), vitality (r = 0.455, *p* < 0.01), and mental health (r = 0.478, *p* < 0.01), thus confirming our third hypothesis that higher resilience scores would correlate with higher scores in each of the dimensions of the SF-36. The results of these correlations are shown in Table 2.

### 3.4. Reliability—Internal Consistency

Cronbach’s alpha was 0.842, indicating good internal consistency. Deleting any one item would lead to a reduction in the estimated Cronbach’s alpha. The item loading values varied between 0.419 and 0.762, the item–total correlation between 0.502 and 0.759, and the communalities between 0.19 and 0.66 (Table 3).

## 4. Discussion

The findings of our study showed that the Portuguese version of the 10-item CD-RISC obtained good psychometric properties and a high level of reliability and validity among young university students, confirming a single factor underlying the 10 elements of the scale present in the different versions validated in other countries.

The mean scores of the 10-item CD-RISC (M = 23.50, SD 6.92) show that the university students in our study have a lower level of resilience compared to other studies that have validated the same version in a population with the same characteristics [3,31,32]. This may be because the study was conducted months after the lockdown restrictions due to the COVID-19 pandemic. As some studies among university students have shown, the conditions experienced during the pandemic have affected health in general. Specifically, to a greater extent, mental health affects the capacity for resilience, as studies carried out in Portugal [33] and other countries have shown [34,35,36]. On the other hand, with respect to sex differences, the male students in our study had statistically significant average scores higher than the women, coinciding with a study carried out among young Spanish university students [3] and with other research that analyzes the influence of sex on resilience among young people [37].

Regarding the factor structure, our results confirm that a single factor underlies resilience building, as in the original version of the 10-item CD-RISC [31] and the validated versions in other countries [3,32,38,39]. This suggests that the 10-item CD-RISC is a one-dimensional measure of resilience, reaffirming the intercultural applications of the instrument by confirming the one-dimensional structure in both the EFA and the CFA, with the latter showing an adjustment index (X2, CFI, TLI, SRMR) very similar in the versions validated for young students [3,32] and a range in agreement with the results obtained in our study.

Regarding convergent validity, our results coincide with previous research in which CDRISC was negatively correlated with constructs such as depression, anxiety [31], and stress [40]. Also, in a university context, our research was in line with the results of a study carried out among young Spanish university students, where reduced states of perceived mental health were associated with reduced levels of resilience [3]. Moreover, convergent validity was also confirmed through positive correlations in the middle range with constructs related to physical, mental, and social well-being and a better self-perception of health, which coincides with some other research carried out [14,32].

Finally, the reliability of the Portuguese version of the 10-item CD-RISC in our study (α = 0.842) was similar but somewhat lower than that of the original version (α = 0.85) [31] and those of other validations carried out among young students (α = 0.85–0.86) [3,32]. 

The psychometric properties of the CD-RISC validated in our study and in other countries among young populations have proven it to be an instrument with adequate measurement invariance, which reflects that it could be considered a gold standard in future research that analyzes resilience.

### 4.1. Limitations

The results should be interpreted with caution given the limitations of this study. The first is that the sample was, for convenience, not representative of the complete Portuguese university context. Second, as a cross-sectional study, the results do not establish predictive validity. However, among the strengths of our study, it should be noted that this is the first validation study of the 10-item CD-RISC for a young Portuguese university population that has shown it to be valid and reliable. It is a short, simple instrument that can be administered efficiently and is suitable for use in community studies.

### 4.2. Involvement for Policy, Practice, and Future Research

The results of this study may be used as a first approach to developing healthy policies and health promotion plans in the university context in which the research was developed, as the average resilience score in our study was lower than the results presented for young university students from other countries. In addition, the 10-item CD-RISC, Portuguese version, for young university students is a short, easy-to-self-administer instrument which can be used as a community screening tool in other settings. Finally, further research needs to be carried out in this field since, as previously explained, this study has certain limitations which need to be improved in future research. For instance, a longitudinal study with a larger, more representative sample could be performed to analyze what elements influence resilience and how we can help to make young university students more resilient people and present an optimal state of mental health, and therefore, good physical and social health.

## 5. Conclusions

The recent events involving the COVID-19 pandemic, lockdown, and social distancing have generated new behaviors among young people, which have negatively affected their mental health. It has been observed that a good level of resilience can be one of the factors that protects young people from poor mental health, so it is crucial to carry out further research analyzing the elements of resilience now that there are instruments available to measure it in a reliable and valid way. In addition, nowadays, shorter scales are required to avoid survey fatigue among participants and to quickly capture the construct to be measured. In conclusion, the current study showed that the Portuguese version of the 10-item CD-RISC for young university students is a brief, valid, and reliable instrument that can be used in future studies to analyze resilience and its relationship with other elements of mental health.

## Figures and Tables

**Table 1:** Average scores of the items, floor effect, and ceiling effect of the 10-item CD-RISC Portuguese version among young university students.

Items	Total Mean (SD)	Men Mean (SD)	Women Mean (SD)	Difference According to Sex (*p*)	U de Mann–Witney	PSest	% Floor Effect	% Celling Effect
1. Able to adapt to change	2.72 (0.91)	3.02 (0.78)	2.62 (0.94)	0.006 *	3175.5	0.38	1.50	20.90
2. Can deal with whatever comes	2.38 (0.93)	2.76 (0.86)	2.25 (0.92)	<0.001 *	2841.5	0.34	3.90	10.20
3. Tries to see the humorous side of problems	2.41 (1.21)	2.91 (1.16)	2.23 (1.17)	<0.001 *	2822	0.34	5.80	23.30
4. Coping with stress can strengthen me	1.99 (1.20)	2.16 (1.13)	1.92 (1.22)	0.242	3725.5	0.45	15.50	11.20
5. Tends to bounce back after illness or hardship	2.50 (1.11)	2.71 (1.15)	2.43 (1.09)	0.122	3589.5	0.43	5.80	18.90
6. Can achieve goals despite obstacles	2.77 (0.99)	2.84 (0.86)	2.75 (1.03)	0.699	4012.5	0.48	1.50	27.70
7. Can stay focused under pressure	2.06 (1.05)	2.45 (0.83)	1.91 (1.09)	0.001 *	2963.5	0.36	6.30	8.30
8. Not easily discouraged by failure	1.94 (1.17)	2.18 (1.23)	1.85 (1.14)	0.066	3479	0.42	12.10	11.20
9. Thinks of self as a strong person	2.45 (1.10)	2.55 (1.03)	2.41 (1.12)	0.556	3938	0.47	5.30	19.40
10. Can handle unpleasant feelings	2.28 (1.06)	2.64 (0.95)	2.15 (1.07)	0.002 *	3034	0.37	4.90	12.60
	**Total** **Mean (SD)**	**Men** **Mean (SD)**	**Women** **Mean (SD)**	**Differences** **According to Sex (*p*)**	**Student’s *t*-Test**	**Cohen’s d**		
10-item CD-RISC	23.50 (6.92)	26.22 (6.05)	22.51 (6.97)	0.001 *	3.495	0.55		

Note: *p*: *p* value (* *p* < 0.05). For the effect sizes, we used PSest (Probability of Superiority) = U/nmen*nwomen: No effect (PSest ≤ 0.0); small (PSest ≥ 0.56); medium (PSest ≥ 0.64); large (PSest ≥ 0.71) (Grissom; 1994 [30]). Cohen’s d: small (d ≤ 0.2), medium (d ≥ 0.5), and large (d ≥ 0.8).

**Table 2:** Pearson’s correlation coefficients between 10-item CD-RISC and DASS-21 and SF-36.

Dimensions	10-Item CD-RISC
Depression (DASS-21)	−0.454 **
Anxiety (DASS-21)	−0.331 **
Stress (DASS-21)	−0.340 **
Physical function (SF-36)	0.175 *
Physical role (SF-36)	0.257 **
Body pain (SF-36)	0.261 **
General health (SF-36)	0.447 **
Vitality (SF-36)	0.455 **
Social function (SF-36)	0.373 **
Emotional role (SF-36)	0.350 **
Mental health (SF-36)	0.478 **

Note: * correlation is significant at the 0.05 level (two-tailed); ** correlation is significant at the 0.01 level (two-tailed).

**Table 3:** Item loadings, item–total correlations, communalities, and Cronbach’s alpha for the 10-item CD-RISC, Portuguese version, among young university students.

Items	Item Loadings (EFA)	Item–Total Correlation	Communalities	Cronbach’s Alpha If the Element Has Been Deleted
1. Able to adapt to change	0.619	0.668	0.41	0.827
2. Can deal with whatever comes	0.705	0.723	0.55	0.82
3. Tries to see the humorous side of problems	0.419	0.502	0.19	0.841
4. Coping with stress can strengthen me	0.476	0.549	0.26	0.837
5. Tends to bounce back after illness or hardship	0.480	0.557	0.26	0.836
6. Can achieve goals despite obstacles	0.696	0.670	0.52	0.822
7. Can stay focused under pressure	0.567	0.631	0.34	0.828
8. Not easily discouraged by failure	0.625	0.652	0.43	0.826
9. Thinks of self as a strong person	0.762	0.759	0.66	0.812
10. Can handle unpleasant feelings	0.675	0.720	0.50	0.819
10-item CD-RISC				0.842

## Data Availability

Data are contained within the article.
